# Challenges of Spa Tourism in Andalusia: Experts’ Proposed Solutions

**DOI:** 10.3390/ijerph18041829

**Published:** 2021-02-13

**Authors:** Rosa Anaya-Aguilar, German Gemar, Carmen Anaya-Aguilar

**Affiliations:** Department of Economics and Business Administration, University of Malaga, Campus El Ejido, s/n, 29071 Malaga, Spain; ranaya@uma.es (R.A.-A.); canaya@uma.es (C.A.-A.)

**Keywords:** spa, Delphi, tourism, health, Andalusia

## Abstract

Water is the common thread and attraction factor of the tourism facilities called “spas”, which are part of health and beauty services. Spa use is currently experiencing a boom that reflects changes in populations, such as an increase in economic wellbeing and a desire to reunite with nature. This research’s objectives were to understand spa tourism’s structural and operational dimensions and to assess this sector’s current situation by using the Delphi method with a panel of 22 experts. The results show that these experts believe that, in Andalusia, spas energize the area as a tourism destination through their natural resources and conservation of key elements. However, spa development policies are scarce, including a lack of autonomous community laws regarding these facilities.

## 1. Introduction

The term “health tourism” is a generalized concept that still needs to be defined in greater detail as it encompasses broad concepts such as spa tourism, wellness, and medical tourism [1]. From health tourism’s demand side, a distinction can be made between wellbeing and healthcare. Wellbeing is pursued only by “healthy” people, with prevention being the main objective. These consumers may seek services similar to those used by clients of normal “cures”, but they participate in this kind of tourism to preserve their health [2].

The experiential value of healthy water-based activities confirms its positive impact on the quality of life, satisfaction and loyalty of people both towards the experience and towards the destination [3].

Spas offer complete destination health resort experiences that promote an improved state of body and mind and high-quality healthy food. Medical spas specifically offer cosmetic surgery procedures and other related services. These places should not be confused with traditional spas that offer preventive medical treatments, such as hydrotherapy [4].

Since ancient times, people have sought hot springs with the idea of improving their health and spas have been born around these springs [5]. Thermal water spas, by definition, are springs of water that flows from the ground naturally, with a high amount of minerals and at a temperature higher than that of the surface by 4 degrees Celsius [6]. These spas have been used since ancient times because these conditions of mineral content and temperature give them therapeutic properties. Although its activity dates back to past centuries [5] and is normally associated with recreation, health and well-being, today, these facilities are also associated with concern for water management [7,8].

The use of spas and their mineral waters has a long tradition over time; however, at present, the Public Health regulations could represent a threat to this practice, since the disinfection processes used in the waters could alter their healing properties [9].

In the 21st century, spas are being reinvented as part of health tourism, based on new concepts and services mainly dominated by people’s desire for wellbeing, especially as expressed by the ideals linked with spas [10,11]. 

Spa users thus can have medical and wellness goals. These two groups have different motivations as members of the former need treatment for medical conditions, but those in the latter group are motivated to take care of their existing health [12]. In response to these demands, the spa sector has grown exponentially in recent decades. Spa facilities have become important sources of income for hotels and resorts, although spa clients prefer good-quality therapies at reasonable prices and with a high level of privacy [13].

**Figure 1:**
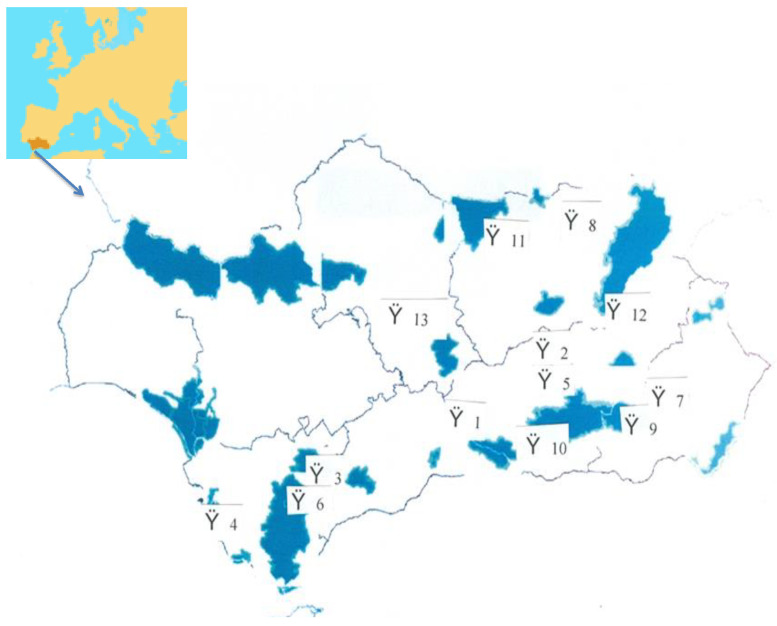
Spas in Andalusia, Spain.

Andalusia, as of 2019, had 13 spas with similar characteristics (see Figure 1) including being places with special water, attractions, legal issues, and adaptations to new trends [14]. This topic has aroused the interest of the authors to better understand the problems of the spa tourism sector. That is why the objective of this research was to know the aspects that favor or hinder the development of spa tourism in Andalusia from the perspective of experts. For this, the Delphi method was used to extract the opinions of a panel of 22 experts.

## 2. Literature Review

Although health tourism is an ancient form of recreation, the role of up-to-date healthcare methods in this form of tourism has been strengthened since 1990, which has sustained the popularity of traditional health services in spas. They help satisfy the demand for healthy lifestyles in which each person is held responsible for maintaining their own health, as well as reducing stress and counteracting recent developments in the “evils” of contemporary societies [15,16]. 

Along with these trends, this sector has also experienced a dynamic increase in its financial importance. The global wellness industry generated 4.5 trillion United States dollars ($) in 2018, growing 6.4% annually from 2015 to 2017 (i.e., from $3.7 trillion to $4.2 trillion), while the global economy grew by only 3.6% annually. The $4.2 trillion spent on wellness expenses account for more than half of the total spent on health worldwide ($7.3 trillion), according to the World Health Organization. 

Specifically, the spa sector in 2017 included more than 149,000 facilities, with $93.6 billion in revenue and 2.5 million workers. The spa sector grew 9.9% annually between 2015 and 2017, and the forecast for 2022 is that earnings will reach $128 billion [17,18]. Additionally, international wellness tourists spend an average of $1528 per trip, 53% more than the typical international tourist. Likewise, national wellness tourists spend $609 per trip, 178% more than the average national tourist [19], although the COVID-19 pandemic has paralyzed the planned goals of wellness tourism for 2022, it is no longer in way to meet that forecast. According to the World Tourism Organization (UNWTO) [20], it will take two to three years for tourism to regain pre-pandemic levels but in the current context of the COVID-19 pandemic, the hydrotherapy sector reinvents its opportunities as alternative tourism away from the masses and claims its sanitary character within society. Many spas have offered their facilities and their medical equipment to the authorities either by accommodating health personnel or transporters, welcoming the elderly or offering their experiences in respiratory treatments of their waters at the service of research [21].

Health tourism has led to a growth in social and economic indicators in the countries that develop it, such as life expectancy, the innovative development of the country, personal spending on health among the population or a reduction in the cost of transportation. In a global context such as the current one, flows of health services are formed from developing countries to developed countries due to innovative solutions in their services and high technology for diagnosis and treatment. There are also flows from developed countries to countries with a developing market for high-quality health services and competitive prices [22].

The benefits derived from health tourism can be analyzed from the concept of value proposition so that the business model includes value captured for the company, value for the client and value for the community and its environment. In order for an organization or company dedicated to health tourism to generate income, it must meet customer expectations and must create value for the community. The expected trend in value creation for the community is proportional to the effectiveness of the influence of customer value after discounting the value captured by the company [23].

The impact of the 2008 economic crisis on tourism was still noticeable when a new crisis appeared caused by the COVID-19 pandemic. This circumstance generates the need to create higher quality tourism products. Health tourism represents an essential element in the creation of a comprehensive tourism product from which many benefits will be obtained: the creation of new jobs, seasonal adjustment of the tourist offer, economic and sociodemographic development of rural areas, satisfaction of needs of tourists of other profiles, and age, among others. That is why it is important to create an attractive offer of health tourism to strengthen the competitiveness of destinations [24].

In recent times, the number of academic research on new factors that attract tourists to spas has increased and shows that demand is significantly dependent on consumers’ choice of particular forms of therapeutic spa treatments based on multiple factors. These variables include age, education, size of tour groups, past spa experiences, type of guest, length of stay, and expected benefits [25,26], as well as feeling of better health intensified by the environment, the relationships with other users and the kindness of the staff that attends them [27]. Other studies also add to this set of variables that influence the demand of tourists for health and social aspects and the desire to explore new places and geological elements that in these places have significant geotourism potential [28].

Spa companies that offer a wide range of services are looking for new customer value propositions to attract fresh market segments as, until now, spas have tended to focus on their natural resources [29].

Research on medical tourism has focused on different topics, including ethical implications and trust issues and spa-related health and wellness services and their quality. Other topics are medical treatments and tourism, sensible practices in medical tourism, medical tourism destinations and their commercialization, and globalization and patients’ internationalization [11].

The components of spa companies’ business model have changed. In the last decade, spas have no longer offered just treatments with accommodation and restaurant services. Currently, leisure activities must be added to enhance the therapeutic and beneficial effects of relaxation services such as stress relief, as well as beauty enhancement, weight loss, fitness, and sports performance. A significant added value is guests’ integration with other people with similar health, lifestyle, and social interests or ways of spending their free time. In this context, new trends have appeared, for example, the need to develop cultural activities, optional childcare during treatments, and introduction of psychologists and social event coordinators [17].

Spa companies need to keep up with changing trends, while some facilities seek to acquire knowledge about clients and their preferences by building relationships with them. In addition to mid-level prices, clients now prefer highly qualified therapists and greater privacy [13]. Hydrotherapeutic techniques use spring water, tap water, or sea water for medicinal purposes together with new technologies, which has rejuvenated the spa sector and allowed it to incorporate a more up-to-date understanding of healthy living [10]. 

The development of new technologies is a key issue, especially those geared toward tourism services and their distribution through mobile devices. To this end, initiatives need to be implemented in this area, such as the use of new technologies to gather valuable data on spa consumers to increase spa destinations’ competitiveness [30,31]. An example of this can be found in spas in Poland that have taken advantage of technologies’ potential to add to their services’ value [29]. The authors of this study found three market segments differentiated by sociodemographic, behavioral, and psychographic factors and argued that this knowledge is important for stakeholders and for tourists themselves, since knowing the needs and motivations of tourists allows better management of the destination.

The role of spas in tourism is seen as increasingly important. Most clients are “core” customers for whom spas and wellness in general are an extremely important part of their lifestyle. Customers’ length of stay can vary greatly along with their needs and expectations. Spas must, therefore, offer facilities for day visitors and short or long stays. 

A major challenge is to capture the interest of men and younger age groups. Beauty, relaxation, or medical conditions are still the main motivations to visit spas, and updates are certainly helping to revalue these ancient healthcare traditions [1], but the clients of spa therapies continue to be mainly older people. Although the current demand for spas reflects heterogeneous needs, spa managers continue to assume that customers have homogeneous demands. One important variable widely acknowledged by management is length of stay [32,33], which is affected by different factors such as age and purchasing power. However, age is undoubtedly the main determinant of length of stay in this type of establishment [34].

In Spain, the Instituto de Mayores y Servicos Sociales (IMSERSO) is an agency of the Spanish social security system responsible for managing complementary social services for the elderly. These services include social tourism and stays in spas for senior citizens. IMSERSO’s Social Thermalism program is financed by the Spanish government. Retirees thus have the opportunity to stay for over a week at a time in spas, where these individuals can receive medical treatments and enjoy leisure activities at a quite low price [35].

The global boom in healthcare has led destinations and companies to specialize in health and wellness tourism as part of their corporate strategy. For some destinations, health tourism can be a way to fight seasonality and diversify within coastal tourism [36]. Tourism has also contributed to the sustainable development of rural destinations. However, tourism’s seasonality intensifies its negative effects, so strategies that fight seasonality generate reliable alternatives that encourage sustainability [37].

In addition, traditional disinfection processes may be incompatible with current health practices, and the question of natural waters’ “untouchability” means that spas’ strategies must be reviewed. These issues highlight the limits of these facilities’ sustainable management [9].

## 3. Materials and Methods

The starting point was the definition of the problems of the SPA, obtained by the authors in interviews with some managers of the spa resorts. At a conceptual level, certain aspects that should be addressed were identified, such as “legal environment”, “natural environment”, “patrimonial environment”, “services: treatments”, “services: human resources”, “services: new technologies”, “facilities and equipment”, “strategies: competitive advantages”, “strategies: competitive position”, and “strategies: image”.

Based on the conceptual approach of the term, a meeting was held between the authors of this study and the Andalusian spa manager in which the different aspects of the environment related to the spa sector in general, services, facilities, and strategies defined in the previous paragraph that could favor or harm the spas and finally the variables that would be part of the questionnaire were decided.

That is why it was decided to develop a pilot questionnaire, which for its validation was submitted to a sample of spa tourism professionals totally independent from the study’s panel of experts, which after some small adaptation was finally used. The final questionnaire consisted of 40 questions measured with a 5-point Likert (where 1 = strongly agree and 5 = strongly disagree) scale that corresponds to the first column of Table 1 and Table 2. In order to facilitate reading, the questions have been grouped under different headings, as seen in the first column of the results tables.

To confirm the questionnaire’s reproducibility and reliability, the diagnostic agreement of the 40 variables included was subjected to a test-retest with a separation of one week. The intraclass correlation coefficients were analyzed and measured using the Landis scale [38]. Values higher than 0.62, 0.62, 0.53, and 0.75 were found for each item contained in the environments, services, facilities and equipment, and strategies sections, respectively (Table 1). 

The questionnaire’s internal consistency was measured using Cronbach’s alpha coefficient, which indicated that there was a high correlation between the items. In the environments section, all values are above 0.667 and, in services, above 0.585. In installations and equipment, the majority are above 0.727, and, in strategies, these values exceed 0.600 in all cases (Table 2).

Next, the team formed by the authors and the head of the Andalusian Association of Thermal Stations chose a panel of experts (Table 3) with some considerations:

The following has been included as an operational definition to be an expert: one person per spa, who had the responsibility of managing it, and with at least two years of seniority in the position or professional performance within the tourist field and university professors specialized in tourism.

The total of experts was 22; 9 internal experts (linked to spa management) and 13 external experts (destination spa professionals but not linked to management. A total of 22 valid questionnaires were received in both the first and second round, which is a response rate of 88%. A consensus of 70% was set as a measure by which to evaluate the investigation’s results (see Table 3).

Once the expert panel was formed (Table 3), the Delphi research method was used. The Delphi technique is defined as “a method of structuring a group communication process that is effective in allowing a group of individuals, as a whole, to deal with a complex problem”.

The experts’ estimates are made in successive rounds, anonymously, in order to try to achieve consensus, but maintaining maximum autonomy on the part of the participants [39].

To implement the method, two circulations of the questionnaire were made: A first round was carried out, and after the collection and statistical analysis of the results, the mean was used as the statistical response of the group, with which all the opinions expressed by the experts were reflected and transmitted to the rest.

The questions posed to the experts are listed in Table 4.

In our study, two rounds were carried out and at the end of the second round the information provided by the experts was collected and tabulated to obtain the mean, standard deviation, and percentage coefficient of variation (Table 5). The standard deviation was estimated to determine the degree of consensus, since the higher the deviation, the lower the consensus in the responses. To compare the consensus difference between the first and second rounds, the percentage coefficient of variation (i.e., standard deviation/mean × 100) was calculated after collapsing the response categories to only “Agree”, “Indifferent”, and “Disagreement”. The values of the first and second rounds were compared, revealing that lower values were obtained for the second round and therefore there was a greater degree of consensus in this round (see Table 5).

## 4. Results

An analysis was conducted using the Mann–Whitney U-test [40] to identify any significant differences in the results by type of specialist. The variable type of mineral-medicinal water (*p* = 0.039) is considered an essential treatment within spa services by all the experts surveyed. Participants see the item that identifies spas with disease (*p* = 0.054) as an imaging strategy based on dissociating spas from disease. The variable of different administrations’ competencies is understood to be a favorable component in spa tourism, along with social event coordinators. The experts consider these actors to be an important differential factor for spas, assigning values with a statistical significance of *p* = 0.161 and *p* = 0.104, respectively (see Table 6).

Based on the consensus measure of 70% established for this research, it was obtained on 30 of the 40 variables proposed (see Table 7). The experts’ opinions did not show a consensus about items such as autonomous community legislation and different administrations’ competencies as favorable aspects of Andalusian spas’ legal environment. However, closeness to a city or the sea is clearly considered a positive factor in spa tourism. Social events coordinators, gyms, and press or souvenir kiosks are seen as basic spa services. In addition, new technologies-based techniques such as electrotherapy are attractive features of spa facilities. 

Experts also agree on improving the image of spas so that it is not linked to disease. The participants additionally consider that preservation of the environment, absence of noise, culture or nature itineraries, monuments, and local customs and culture will attract clients and help develop spa tourism further. Treatments based on the type of medicinal mineral water and a variety of health treatments are other essential features of successful spas. 

The results also reveal that spa staff’s qualifications and training and a variety of specialists, as well as the technological evolution of physiotherapy, kinesiotherapy, and osteopathy, can be differentiating factors for spas. Regarding facilities, entertainment and leisure activities spaces and rooms, leisure and entertainment areas for children, and spas’ internal environments (i.e., lighting, decoration, and music) are considered essential. The experts further think that competitive advantages can come from using new technologies in commercialization, marketing packages through travel agencies, maintaining tourist loyalty policies, and exploiting the “spa” brand as a synonym of quality. Other advantages can be gained from deseasonalizing offers and making the most of the boom in health tourism. Finally, the specialists surveyed agree that identifying spas with tourism, recreation, relaxation, and beauty will enhance their competitiveness.

## 5. Discussion

This work presents as a limitation the elaboration of a specific questionnaire for the study; however, based on what was provided by the group of experts for its elaboration (note that this group is not the same as that of respondents to the study), we proceeded to its validation by means of the Cronbach’s alpha test and the Intraclass Correlation Coefficient.

To compensate for the methodological limitations that some authors attribute to the Delphi technique [41,42,43], the current study carried out this stage of the Delphi technique meticulously and with maximum rigor, seeking to obviate or minimize this limitation in order to identify the consensus correctly. Twenty-five experts were selected initially, and a total of 22 valid questionnaires were received. Thus, participation was 22 experts, which substantially exceeded the minimum of seven experts suggested by Rand Corporation researchers [44]. The response rate in the present research’s first and second rounds was 88%.

Overall, the experts suggest that the current legal environment creates difficulties for spas’ development due to the competencies shared between ministries (i.e., tourism and health) and a lack of specific—and thus problematic—autonomous community legislation. However, with respect to the natural environment, the participants agree that this factor favors spa tourism, although many clients are indifferent to proximity to the sea or a city. These results corroborate the findings of other studies [5] that have focused on mineral springs’ value based on their ability to attract tourists or their surrounding environment. 

As some authors argue, natural and water-based experiences create loyalty to both the type of experience and the tourist destination [3]. The attraction exerted by Andalusian spas confirms the growth trend of the spa sector at an international level [17,45]; although at the moment the COVID-19 pandemic has paralyzed world tourism [20], the hydrothermal sector is reinventing itself offering its facilities and medical equipment at the service of public health [21].

Regarding the spas’ heritage environment, the experts’ responses show a consensus on this variable as a positive factor in spa tourism since this element is an added value with the power to attract more users. This aspect correlates positively with other research that contributes that the model of spa companies has changed in recent times, offering something more than accommodation and restaurant. Thus, new trends also offer other ways of spending free time such as cultural activities, among others [17]. However, the specialists surveyed are less favorable about complementary activities, for example, press or souvenir kiosks, but the respondents underline the importance of applying new technologies in these activities.

Within the services variables, all the participants see the type of medicinal mineral water as a further favorable aspect of spa tourism. Other aspects, such as the variety of treatments offered and innovations in these, are indicated as the best path to follow in commercialization. Human resources qualifications, specializations, and continuous training in this specific sector are considered a line of strategy to continue into the future.

These results are in line with other research that adds that spa companies continually seek new value propositions for the customer with the idea of attracting new market segments [11,13,29].

New technologies’ applications are tools that clearly need to be exploited by the sector. Similar conclusions were drawn by researchers in a study on spas in Poland who found that new technologies are key both for collecting valuable data on spa consumers and for adding value to their services [29,30,31].

In terms of facilities and equipment, the results also show a high level of consensus. Of the variables analyzed, the experts’ assessment indicates that spas need to invest in improving specific components of their treatment facilities and activities, such as water circuits, health and beauty treatment areas, and the internal environment. However, other studies indicate that the attraction to spas is exerted more by other factors such as the landscape, social relationships, or the possibility of other activities [27,28].

The responses to the strategy items in this survey show that there is a strong consensus among spa experts, who perceive these strategies as producing competitive advantages. Specific strategies include both government aid for spas that want to apply new technologies and packages marketed through travel agencies and the internet. Other strategies are user loyalty programs, a combined brand of “Andalusian spas” as a synonym for quality, seasonal adaptation, and advantages derived from the boom in health tourism. However, as some authors maintain [14], Andalusian spas have a certain delay in the adoption of updated therapies and other studies indicate that although they have made an effort to modernize the facilities, these are not entirely sufficient [46].

All the respondents agree that new forms of commercialization and technologies enhance spas’ ability to take advantage of recent trends in world tourism [26]. Health tourism’s growth, new technologies’ support, and fresh approaches to necessary changes are trends that need to be harnessed to develop this sector, in line with what other researchers and international institutes detail [16,17,19,20,26]. The surveyed experts’ ideas coincide with some researchers’ [26,29] work on developing new business models adapted to market segments with more income, as well as those willing to spend money on themselves.

The present results reveal a lack of consensus on whether a strategy needs to be implemented to prevent the popular imagination from identifying spas with disease or larger populations, which other researchers have also confirmed [46]. In Spain, Social Security finances remain for the elderly through the thermalism program [35], but in addition, the experts in spas surveyed in this study seem to be immersed in an important debate on the IMSERSO Social Thermalism program, as some respondents see this as a market intervention and price control. The aging of the population implies a growing potential demand for health services and shorter and more fragmented vacations, while younger clients are another segment to target. However, different studies indicate that it is a major challenge for spa companies to capture the interest of younger groups [1] and recognize that the length of stay is greatly affected by age and purchasing power [33,34,47].

Regarding marketing, the experts agree that this should be developed further through the internet or travel agencies. Studies conducted decades ago also highlighted a lack of product promotion in the spa sector [46,48]. As mentioned previously, the present results indicate no agreement has been reached on the need to develop strategies for disassociating spas’ image from elderly age groups and sickness. Previous research [48] has found that 70% of the Spanish population thinks that the type of people likely to use spas are older, sick people or that these facilities still have a more traditional image synonymous with old age. Therefore, spas are perceived as offering limited recreation and even being monotonous and boring [49]. Notably, studies in other countries do not confirm these findings, as spas are an attractive, strategically placed product [34,50]. (BOE, 1973). However, the international trend shows a broad growth of health and wellness tourism [17,45], with a high demand for healthy styles [15,16], and that people have searched for thermal springs in ancient times [5] which, due to their mineralization and temperature characteristics, gives them a special added value [6].

## 6. Conclusions

Various competencies are shared between the different entities involved in spa development, including tourism, health, and local administrations. In addition, a full development of autonomous community laws regarding spas is lacking in Andalusia, in contrast to Galicia, Cantabria, or Extremadura. The experts surveyed for this study believe that these conditions do not favor spa tourism in the region in question.

Natural resources, such as the type of medicinal mineral water and conservation of the surrounding areas’ natural environment and socio-cultural heritage, are seen as essential elements of spa tourism. Conversely, an inadequate protection of and unsustainable development in these environments are among the most important problems. Policies designed to guide this type of tourism’s expansion are still scarce in Andalusia. Spas have also tried on many occasions to reduce their dependence on IMSERSO’s Social Thermalism program by expanding their offer in order to attract other segments of tourists and weaken many people’s associations of disease and elderly age groups with spa tourism.

In the questionnaire, the variable of water circuits is listed under new technologies rather than with other installations, but some experts believe they should be put in the installations category because these circuits have a more playful component. Installations contains similar leisure elements, while more clinical variables have been included in a different section. Thus, this question could be of interest to further investigations.

This research has a limitation, and that is that it only includes experts from the Andalusia region. However, we understand that this limitation is offset by the deep knowledge of the sector that the experts have in years of experience both in management and in university teaching, so the study could be extrapolated to any point in Spain and even to countries with similar climatic conditions.

The study presents another limitation, as the concept of problem could have been approached from a multidimensional perspective, that is, that this concept included several dimensions. This would be desirable, but for this, a larger sample size of experts would be desirable, or possibly an approach more like a quantitative technique than a Delphi study, which is basically a qualitative technique suggesting possible lines of research in the future.

## Figures and Tables

**Table 1:** Reliability of the 40 items of the Delphi questionnaire. Test-retest (one week apart) (*n* = 6 ^a^).

	Items	CCI ^b^
	**Legal environment**	
1	Water law	0.97
2	Regional legislation	0.92
3	Competences of the different Administrations	0.91
	**Natural environment**	
4	Weather	0.62
5	Close to the sea	0.92
6	Close to the city	0.73
7	Conservation of the environment	0.88
8	Little urban pressure	0.71
9	Absence of noise	0.62
10	Access infraestructures	0.57
11	Cultural of nature itineraries	1.00
	**Heritage environment**	
12	Monuments	0.50
13	Local customs and culture	0.60
	**Services: treatements**	
14	Type of mineral-medicinal water	0.41
15	Variety of health treatments	1.00
16	Variety of beauty treatments	1.00
17	Innovative treatments	0.83
	**Services: human resources**	
18	Qualification and training	0.88
19	Variety of specialists (doctors, physiotherapists, masseurs, sports professionals	0.88
20	Entertainers	0.57
	**Services: new technologies**	
21	Water circuits	0.62
22	Electrotherapy	0.41
23	Physiotherapy, kinesitherapy and osteopathy	0.71
24	Health and beauty	0.80
	**Facilities and equipment**	
25	Gym zone	1.00
26	Spaces and rooms for entertainment and recreational activities	0.92
27	Newsstands and souvenirs	1.00
28	Area for physiotherapy, kinesitherapy and osteopathy	0.62
29	Leisure and entertainment area for children	1.00
30	Internal environment of the center (lighting, decoration, music)	1.00
	**Strategies: competitive advantages**	
31	Application of new technologies for commercialization	0.43
32	Marketing packages through travel agencies	0.76
33	Tourist loyalty policy	0.83
34	Use of the ‘Spas’ brand as a synonym for quality	0.79
35	Seasonally adjusted	0.82
36	Taking advantage of the boom in health tourism	1.00
	**Competitive position strategies**	
37	Identification of spa with tourism, recreation and relaxation	0.79
38	identification of spa with beauty.	0.82
	**Strategies: new image**	
39	Identification of spa with older population	0.59
40	identification of spa with disease	0.75

^a^ Corresponds to 6 people, 2 men and 4 women, with ages between 30 and 46 years of age and different professions not belonging to the main sample. ^b^ Intraclass correlation coefficient between cases with valid response. It can be assessed with the Landis and Koch scale (38): <0.00: Poor; 0.00–0.20: Slight; 0.21–0.40: Fair; 0.41–0.60: Moderate; 0.61–0.80: Substantial; 0.81–1.00: Almost perfect.

**Table 2:** Internal reliability of the questionnaire (Cronbach’s alpha) (*n* = 6 ^a^).

		Alfa de Cronback
	**Legal environmental**	
1	Water law	0.984
2	Regional legislation	0.960
3	Competences of the different Administrations	0.952
	**Natural environment**	
4	Weather	0.762
5	Close to the sea	0.960
6	Close to the city	0.844
7	Conservation of the environment	0.935
8	Little urban pressure	0.828
9	Absense of noise	0.762
10	Access infraestructures	0.727
11	Cultural of nature itineraries	1.000
	**Heritage environment**	
12	Monuments	0.667
13	Local customs and culture	0.750
	**Services: treatements**	
14	Type of mineral-medicinal water	0.585
15	Variety of health treatments	1.000
16	Variety of beauty treatments	1.000
17	Innovative treatments	0.906
	**Services: human resources**	
18	Qualification and training	0.935
19	Variety of specialists (doctors, physiotherapists, masseurs, sports professionals)	0.935
20	Enterteiners	0.724
	**Services: new technologies**	
21	Water circuits	0.762
22	Electroterapy	0.585
23	Physioterapy, kinesitherapy and osteopathy	0.828
24	Health and beauty	0.800
	**Facilityes and equipment**	
25	Gym zone	0.375
26	Spaces and rooms for entertainment and recreational activities	0.750
27	Newsstands and souvenirs	1.000
28	Area for physiotherapy, kinesitherapy and osteopathy	0.762
29	Leisure and entertainment area for children	0.906
30	Internal environmental of the center (lighting, decoration, music)	1.000
	**Strategies: competitive advantages**	
31	Application of new technologies for commercialization	0.600
32	Marketing packages through travel agencies	0.861
33	Tourist loyalty policy	0.906
34	Use of the ‘Spas’ brand as a synonym for quality	0.882
35	Seasonally adjusted	0.928
36	Taking advantage of the boom in health tourism	1.000
	**Competitive position strategies**	
37	Identification of spa with tourism, recreation and relaxation	0.727
38	Identification of spa with beauty	0.822
	**Strategies: new image**	
39	Identification of spa with older population	0.744
40	Identification of spa with disease	0.860

^a^ corresponds to 6 people, 2 men and 4 women, with ages between 30 and 46 years of age and different professions no belonging to the main sample.

**Table 3:** Experts’ professional activity and experience in thermal tourism.

Expert Number	Experts’ Professional Duties at Time of Delphi Study	Experience in Years
1	Managing director of spa	10
2	Spa manager	10
3	Spa owner	10
4	Managing director of spa	15
5	Head of spa quality	10
6	Managing director of spa	10
7	Manager of tourism destination with a spa resort	8
8	Expert in medicinal mineral waters	10
9	Tourism destination spa informant	10
10	Tourism destination spa informant	10
11	Tourism expert	7
12	Tourism expert	8
13	Tourism destination spa informant	9
14	Tourism expert	10
15	Tourism expert	10
16	Municipal manager of spa destination	8
17	Municipal manager of spa destination	8
18	Managing director of spa	15
19	Head of spa quality	10
20	Managing director of spa	15
21	Head of spa administration	15
22	Tourism expert	6

**Table 4:** Questions posed to the experts.

Question Block of the Questionnaire	Question Asked
Legal environment	“if the current situation of the spa in terms of different administrative elements is favored by the legal environment”. (Items 1–3)
Natural environment	“if the natural environment of the spa destination will act as an attractive factor in spa tourism”. (Items 4–11)
Heritage environment	“if the heritage environment where the spa is located will act as an attractive factor in spa tourism”. (Items 12 and 13)
Services: treatments	“if the treatments are essential elements of the services offered by the spas”. (Items 14–17)
Services: human resources	“if human resources are essential and indispensable elements of the services offered by spas”. (Items 18–20)
Services: new technologies	“if the new technologies applied to the services offered will act as an attractive factor in spa tourism”. (Items 21–24)
Facilities and equipment	“if the items contained in questions 25 to 30 are essential elements of spa tourism”. (Items 25–30)
Strategies: competitive advantages	“if the items contained in questions 31–36 will be factors to obtain competitive advantages”.
Strategies: competitive position	“if the items contained in questions 37 and 38 will place the spas in a competitive position”.
Strategies: image	“if the items contained in questions 39 and 40 would be necessary to change the image of those aspects”.

**Table 5:** Dispersion of criteria of experts in thermal tourism in first and second rounds (*n* = 22) ^a.^

Variable	First Round Variation Coefficient (%)	Second Round Variation Coefficient (%)
**Legal environment**		
1. Water laws	52.8	47.3
2. Autonomous community legislation	39.7	48.4
3. Different administrations’ competencies	46.4	47.3
**Natural environment**		
4. Climate	41.2	41.2
5. Closeness to the sea	39.3	36.6
6. Closeness to a city	42.4	37.9
7. Conservation of the environment	0.0	0.0
8. Little urban pressure	26.6	26.6
9. Absence of noise	20.0	20.0
10. Access to infrastructure	30.7	30.7
11. Culture or nature itineraries	0.0	0.0
**Heritage environment**		
12. Monuments	20.0	20.0
13. Local customs and culture	20.0	20.0
**Services: treatments**		
14. Type of medicinal mineral water	20.0	20.0
15. Variety of health treatments	0.0	0.0
16. Variety of beauty treatments	33.0	33.0
17. Innovative treatments	33.0	33.0
**Services: human resources**		
18. Qualifications and training	0.0	0.0
19. Variety of specialists	0.0	0.0
20. Social events coordinators	44.7	42.8
**Services: new technologies**		
21. Water circuits	21.0	21.0
22. Electrotherapy	44.7	48.6
23. Physiotherapy, kinesiotherapy, and osteopathy	28.2	28.2
24. Health and beauty procedures	42.1	42.1
**Installations and equipment**		
25. Gym area	46.2	42.8
26. Spaces and entertainment, activity, and play areas	43.3	43.3
27. Press and souvenir kiosks	51.6	45.5
28. Physiotherapy, kinesiotherapy, and osteopathy areas	27.3	27.3
29. Children’s leisure and entertainment areas	35.0	35.0
30. Internal environment: lighting, decoration, and music	0.0	26.6
**Strategies: competitive advantages**		
31. Application of new technologies for commercialization	26.6	26.6
32. Packages marketed through travel agencies	26.6	26.6
33. Tourist loyalty policy	20.0	20.0
34. Use of “spa” brand as synonym of quality	26.6	26.6
35. Deseasonalization	41.2	41.2
36. Taking advantage of health tourism boom	20.0	20.0
**Strategies: competitive position**		
37. Identification of spas with tourism, leisure, and relaxation	0.0	0.0
38. Identification of spas with beauty	43.1	43.1
**Strategies: image**		
39. Identification of spas with a larger population	51.0	43.7
40. Identification of spas with disease	49.3	43.8

^a^ percentage variation coefficient = standard deviation/mean × 100, after collapsing response categories into “Agreement”, “Indifferent”, and “Disagreement”. If the value is greater than 50%, it is usually interpreted as a sign that no agreement has been reached.

**Table 6:** Results of experts in thermal tourism in the second round according to type of expert (*n* = 22) ^a^.

Variable	Internal Expert (Number = 9)		External Expert (Number = 13)		*p*-Value ^b^
	Number	Mean ± SD ^b^	Number	Mean ± SD ^b^	
**Legal environment**					
1. Water laws	6	2.33 ± 1.03	8	1.87 ± 0.64	0.387
2. Autonomous community legislation	5	2.60 ± 1.14	9	2.33 ± 0.86	0.573
3. Different administrations’ competencies	6	3.33 ± 1.36	10	2.50 ± 0.97	0.161
**Natural environment**					
4. Climate	9	1.44 ± 0.72	13	1.84 ± 1.06	0.281
5. Closeness to the sea	8	3.00 ± 1.51	12	2.41 ± 0.79	0.307
6. Closeness to a city	8	2.75 ± 1.16	12	2.91 ± 0.90	0.933
7. Conservation of the environment	9	1.22 ± 0.44	13	1.23 ± 0.43	0.963
8. Little urban pressure	9	1.33 ± 0.50	13	1.61 ± 0.76	0.421
9. Absence of noise	9	1.22 ± 0.44	13	1.38 ± 0.65	0.606
10. Access to infrastructure	9	1.66 ± 0.70	13	1.53 ± 0.77	0.576
11. Culture or nature itineraries	9	1.33 ± 0.50	13	1.61 ± 0.50	0.203
**Heritage environment**					
12. Monuments	9	1.33 ± 0.50	13	1.61 ± 0.65	0.304
13. Local customs and culture	9	1.44 ± 0.52	13	1.69 ± 0.63	0.365
**Services: treatments**					
14. Type of medicinal mineral water	9	1.00 ± 0.00	13	1.46 ± 0.66	0.039
15. Variety of health treatments	9	1.11 ± 0.33	13	1.23 ± 0.43	0.484
16. Variety of beauty treatments	9	1.77 ± 0.66	13	1.69 ± 0.85	0.664
17. Innovative treatments	9	1.77 ± 0.83	13	1.61 ± 0.76	0.635
**Services: human resources**					
18. Qualifications and training	9	1.11 ± 0.33	13	1.07 ± 0.27	0.788
19. Variety of specialists	9	1.22 ± 0.44	13	1.30 ± 0.48	0.665
20. Social events coordinators	8	1.75 ± 1.16	12	2.33 ± 0.65	0.104
**Services: new technologies**					
21. Water circuits	9	1.33 ± 0.50	11	1.45 ± 0.68	0.785
22. Electrotherapy	8	2.62 ± 1.30	11	2.27 ± 0.46	0.736
23. Physiotherapy, kinesiotherapy, and osteopathy	9	1.55 ± 0.72	11	1.45 ± 0.68	0.727
24. Health and beauty procedures	9	1.44 ± 1.01	12	1.33 ± 0.65	0.961
**Installations and equipment**					
25. Gym area	8	2.50 ± 0.92	12	2.16 ± 0.57	0.344
26. Spaces and entertainment, activity, and play areas	9	1.77 ± 0.83	13	2.07 ± 0.86	0.430
27. Press and souvenir kiosks	8	2.62 ± 0.74	12	2.75 ± 0.96	0.899
28. Physiotherapy, kinesiotherapy, and osteopathy areas	9	1.33 ± 0.50	12	1.50 ± 0.79	0.797
29. Children’s leisure and entertainment areas	9	2.22 ± 0.44	13	2.00 ± 0.70	0.434
30. Internal environment: lighting, decoration, and music	9	1.55 ± 0.72	13	1.46 ± 0.66	0.759
**Strategies: competitive advantages**					
31. Application of new technologies for commercialization	9	1.66 ± 0.70	13	1.46 ± 0.66	0.452
32. Packages marketed through travel agencies	9	1.44 ± 0.72	13	1.38 ± 0.65	0.870
33. Tourist loyalty policy	9	1.11 ± 0.33	13	1.30 ± 0.63	0.455
34. Use of “spa” brand as synonym of quality	9	1.11 ± 0.33	13	1.46 ± 0.77	0.254
35. Deseasonalization	9	1.22 ± 0.44	13	1.61 ± 0.96	0.349
36. Advantage taken of health tourism boom	9	1.00 ± 0.00	13	1.23 ± 0.59	0.228
**Strategies: competitive position**					
37. Identification of spas with tourism, leisure, and relaxation	9	1.11 ± 0.33	13	1.15 ± 0.37	0.779
38. Identification of spas with beauty	9	1.55 ± 0.88	13	1.84 ± 0.89	0.362
**Strategies: image**					
39. Identification of spas with larger populations	8	2.37 ± 0.51	12	2.25 ± 1.05	0.403
40. Identification of spas with disease	7	2.71 ± 0.75	12	2.08 ± 0.51	0.054

^a^: Internal expert is the one who is part of the spa, and external a specialist in university teaching tourism specialized in tourism disciplines. ^b^: Mann-Whitney test.

**Table 7:** Results for experts in spa tourism in first and second rounds (number = 22) ^a^.

Variable	First Round		Second Round	
	Number	Mean ± SD ^a^	Number	Mean ± SD
**Legal environment**				
1. Water laws	15	2.00 ± 1.07	14	2.07 ± 0.83
2. Autonomous community legislation	16	2.81 ± 1.17	14	2.43 ± 0.94
3. Different administrations’ competencies	17	2.88 ± 1.32	16	2.81 ± 1.17
**Natural environment**				
4. Climate	22	1.68 ± 0.95	22	1.68 ± 0.95
5. Closeness to the sea	22	2.82 ± 1.30	20	2.65 ± 1.14
6. Closeness to a city	22	2.86 ± 1.17	20	2.85 ± 0.99
7. Conservation of the environment	22	1.23 ± 0.43	22	1.23 ± 0.43
8. Little urban pressure	22	1.50 ± 0.67	22	1.50 ± 0.67
9. Absence of noise	22	1.32 ± 0.57	22	1.32 ± 0.57
10. Access to infrastructure	22	1.59 ± 0.73	22	1.59 ± 0.73
11. Culture or nature itineraries	22	1.50 ± 0.51	22	1.50 ± 0.51
**Heritage environment**				
12. Monuments	22	1.50 ± 0.60	22	1.50 ± 0.60
13. Local customs and culture	22	1.59 ± 0.59	22	1.59 ± 0.59
**Services: treatments**				
14. Type of medicinal mineral water	22	1.27 ± 0.55	22	1.27 ± 0.55
15. Variety of health treatments	22	1.18 ± 0.39	22	1.18 ± 0.39
16. Variety of beauty treatments	22	1.73 ± 0.77	22	1.73 ± 0.77
17. Innovative treatments	22	1.68 ± 0.78	22	1.68 ± 0.78
**Services: human resources**				
18. Qualifications and training	22	1.09 ± 0.29	22	1.09 ± 0.29
19. Variety of specialists	22	1.27 ± 0.46	22	1.27 ± 0.46
20. Social events coordinators	22	2.18 ± 1.01	20	2.10 ± 0.91
**Services: new technologies**				
21. Water circuits	20	1.40 ± 0.60	20	1.40 ± 0.60
22. Electrotherapy	17	2.47 ± 1.07	19	2.42 ± 0.90
23. Physiotherapy, kinesiotherapy, and osteopathy	20	1.50 ± 0.69	20	1.50 ± 0.69
24. Health and beauty procedures	21	1.38 ± 0.80	21	1.38 ± 0.80
**Installations and equipment**				
25. Gym area	22	2.27 ± 0.88	20	2.30 ± 0.73
26. Spaces and entertainment, activity, and play areas	22	1.95 ± 0.84	22	1.95 ± 0.84
27. Press and souvenir kiosks	22	2.50 ± 1.01	20	2.70 ± 0.86
28. Physiotherapy, kinesiotherapy, and osteopathy areas	21	1.43 ± 0.68	21	1.43 ± 0.68
29. Children’s leisure and entertainment areas	22	2.09 ± 0.61	22	2.09 ± 0.61
30. Internal environment: lighting, decoration, and music	22	1.50 ± 0.67	22	1.50 ± 0.67
**Strategies: competitive advantages**				
31. Application of new technologies for commercialization	22	1.55 ± 0.67	22	1.55 ± 0.67
32. Packages marketed through travel agencies	22	1.41 ± 0.67	22	1.41 ± 0.67
33. Tourist loyalty policy	22	1.23 ± 0.53	22	1.23 ± 0.53
34. Use of “spa” brand as synonym of quality	22	1.32 ± 0.65	22	1.32 ± 0.65
35. Deseasonalization	22	1.45 ± 0.80	22	1.45 ± 0.80
36. Advantage taken of health tourism boom	22	1.14 ± 0.47	22	1.14 ± 0.47
**Strategies: competitive position**				
37. Identification of spas with tourism, leisure, and relaxation	22	1.14 ± 0.35	22	1.14 ± 0.35
38. Identification of spas with beauty	22	1.73 ± 0.88	22	1.73 ± 0.88
**Strategies: image**				
39. Identification of spas with larger populations	22	2.36 ± 1.26	20	2.30 ± 0.86
40. Identification of spas with disease	22	2.41 ± 1.01	19	2.32 ± 0.67

^a^ The comparison of variances (standard deviation squared) between the two rounds was not statistically significant for any variable according to the contrast with the Snedecor F, which indicates that for all variables, considering the stop criterion, we consider the process in the second round.

## Data Availability

The data presented in this study are available on request from the corresponding author.
